# Multifaceted Role of the Urokinase-Type Plasminogen Activator (uPA) and Its Receptor (uPAR): Diagnostic, Prognostic, and Therapeutic Applications

**DOI:** 10.3389/fonc.2018.00024

**Published:** 2018-02-12

**Authors:** Niaz Mahmood, Catalin Mihalcioiu, Shafaat A. Rabbani

**Affiliations:** ^1^Department of Medicine, McGill University Health Centre, Montreal, QC, Canada; ^2^Department of Oncology, McGill University Health Centre, Montreal, QC, Canada

**Keywords:** uPA, urokinase-type plasminogen activator receptor, plasminogen activator system, plasminogen activator inhibitor-1, PAI-2, ATN-658, metastasis, cancer imaging

## Abstract

The plasminogen activator (PA) system is an extracellular proteolytic enzyme system associated with various physiological and pathophysiological processes. A large body of evidence support that among the various components of the PA system, urokinase-type plasminogen activator (uPA), its receptor (uPAR), and plasminogen activator inhibitor-1 and -2 (PAI-1 and PAI-2) play a major role in tumor progression and metastasis. The binding of uPA with uPAR is instrumental for the activation of plasminogen to plasmin, which in turn initiates a series of proteolytic cascade to degrade the components of the extracellular matrix, and thereby, cause tumor cell migration from the primary site of origin to a distant secondary organ. The components of the PA system show altered expression patterns in several common malignancies, which have identified them as ideal diagnostic, prognostic, and therapeutic targets to reduce cancer-associated morbidity and mortality. This review summarizes the various components of the PA system and focuses on the role of uPA–uPAR in different biological processes especially in the context of malignancy. We also discuss the current state of knowledge of uPA–uPAR-targeted diagnostic and therapeutic strategies for various malignancies.

## Introduction

Tumor metastasis is a multistep process initiated when cancer cells acquire the ability to invade the surrounding matrix and migrate to seed distant organs *via* hematogenous or lymphatic routes (1). Almost 90% of the cancer-related deaths in human are caused due to the metastatic spread of the tumor cells (2, 3). Even though therapeutic strategies targeting the primary tumors have been improved markedly over the years, targeting tumor metastasis has only seen a minimal to modest success. Since the pathogenesis of metastasis involves a series of sequential events regulated by different molecular determinants, it stands to reason that therapeutic modalities targeting the key molecules and signaling pathways involved in the metastatic cascade may serve as an effective therapeutic strategy to block cancer progression.

One of the major events that underlie metastasis is the proteolytic degradation of the extracellular matrix (ECM) to promote tumor cell invasion, migration, and homing to distant organs (4). Even though several protease systems are implicated in this process, a large body of evidence identified the uPA–urokinase-type plasminogen activator receptor (uPAR) system as a central player in mediating proteolysis during cancer invasion and metastasis (5, 6). Further studies have indicated that the functionality of the uPA–uPAR system is not only limited to proteolysis. In fact, the present consensus suggests that the uPA–uPAR system plays a broader role in multiple stages of cancer starting from tumorigenesis to metastasis (6, 7). Elevated expression of the components of the uPA–uPAR system has been shown to be related to adverse patient outcomes in different types of cancer (8–12). As such, the components of the uPA–uPAR system have been identified as excellent candidates for anticancer therapies (13, 14). This review is aimed to summarize our current knowledge on the role of the uPA–uPAR system in cancer.

## The Plasminogen Activator (PA) System

The PA system was initially thought to play a role in the dissolution of clots formed by the fibrins (15). However, later studies have demonstrated that the PA system has additional functions in other biological processes such as embryogenesis, angiogenesis, cell migration, wound healing, inflammatory response, as well as apoptotic cell death (15). In cancer, the PA system plays a dominant role in tumor growth, angiogenesis, tumor cell invasion, migration, and metastasis.

**Figure 1:**
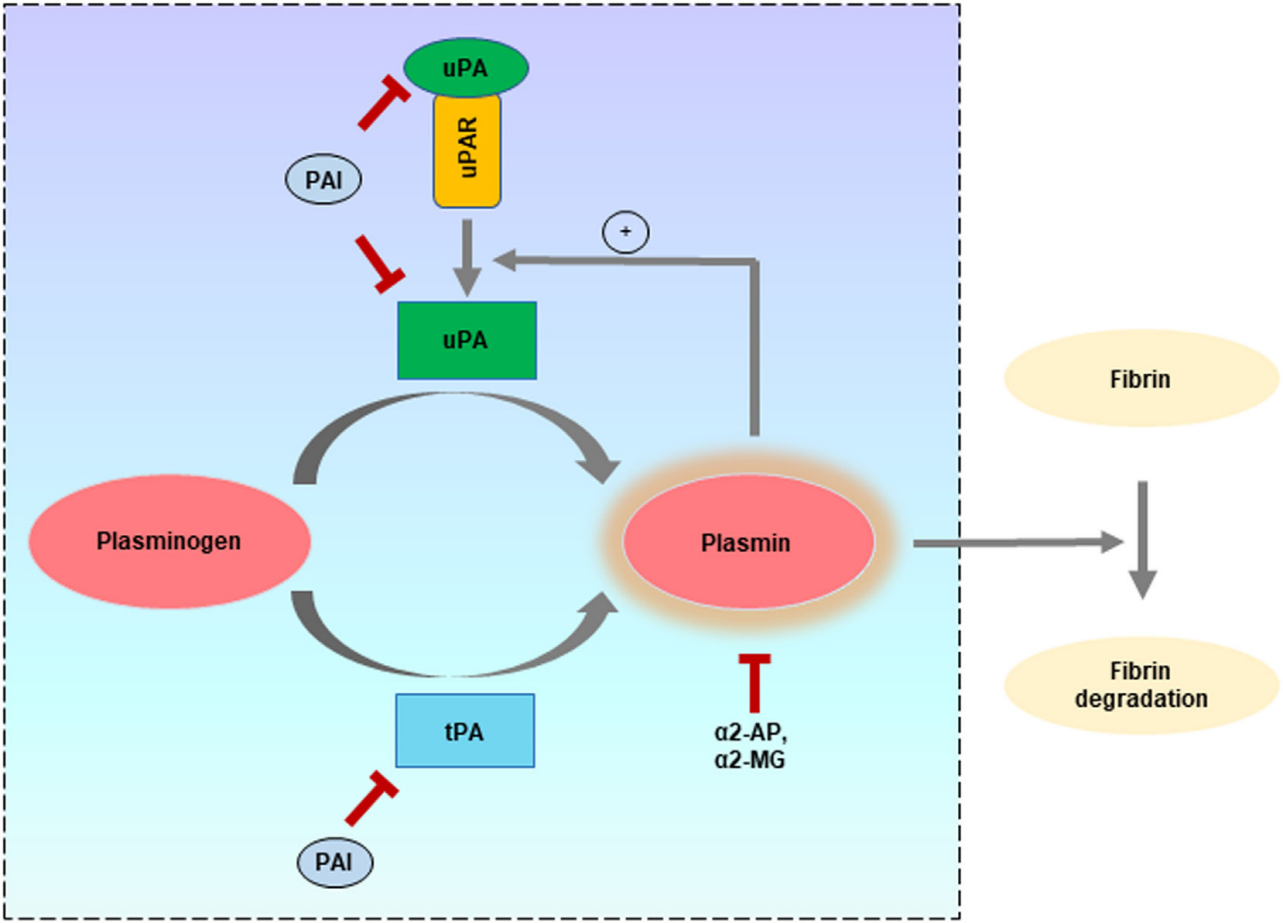
Different components of the plasminogen activator (PA) system and role in fibrinolysis. Schematic representation of the ability of type plasminogen activator (tPA) and uPA to independently activate “plasminogen” to form the active proteolytic enzyme “plasmin” which can mediate fibrinolysis to keep the blood free from clotting. In addition to their fibrinolytic effects, tPA and uPA are implicated in many other physiological and pathophysiological processes. Both tPA and uPA can be inhibited by plasminogen activator inhibitors (PAI) such as plasminogen activator inhibitor-1 and PAI-2, while plasmin can be inhibited by α2-antiplasmin (α2-AP) and α2-macroglobulin (α2-MG). The different components belonging to the PA system are enclosed within the square region.

The major physiological function of the PA system is to convert the inactive plasminogen to plasmin, which can be mediated by two types of PAs: the tissue type plasminogen activator (tPA) and uPA. Different inhibitory proteins have also been identified, which primarily regulate the plasminogen activation by both tPA and uPA. Among these, the two well-characterized endogenous inhibitors of tPA and uPA are plasminogen activator inhibitor-1 (PAI-1) and PAI-2. On the other hand, plasmin activation is regulated by inhibitors such as α2-antiplasmin and α2-macroglobulin (Figure 1).

The activation of plasminogen by tPA and uPA is under temporal and spatial regulation (16). tPA is mainly synthesized by the endothelial cells and functions in clot lysis (17). uPA can also function to protect from the deposition of fibrin and has been used as fibrinolytic/thrombolytic agent (18). Even though both tPA and uPA are present in tumor cells, uPA is more commonly associated with cancer progression (19). For this distinct role, much attention has been given to understand the functionality of the uPA–uPAR system in cancer, which has led to its identification, characterization, and validation as a prognostic, diagnostic, and therapeutic target in several common cancers (18, 20–22). In the following section, the structure and functional significance of uPA, uPAR, and the two inhibitors (PAI-1 and PAI-2) are briefly discussed.

### Urokinase-Type Plasminogen Activator (uPA)

The “Urokinase-type Plasminogen Activator” or simply “urokinase” (uPA) is a key serine protease involved in the conversion of inactive plasminogen into active plasmin, which in turn functions in a range of events of the metastatic cascade (23). It was first identified in the urine in 1947 by MacFarlane and Pilling who reported on the fibrinolytic activity of a novel “unnamed” protein (24). Half a decade later, Sobel and colleagues named this “unnamed” protein as “urokinase” (25). Further studies have reported the presence of urokinase in plasma, seminal fluid, and the ECM of many tissues (23, 26).

**Figure 2:**
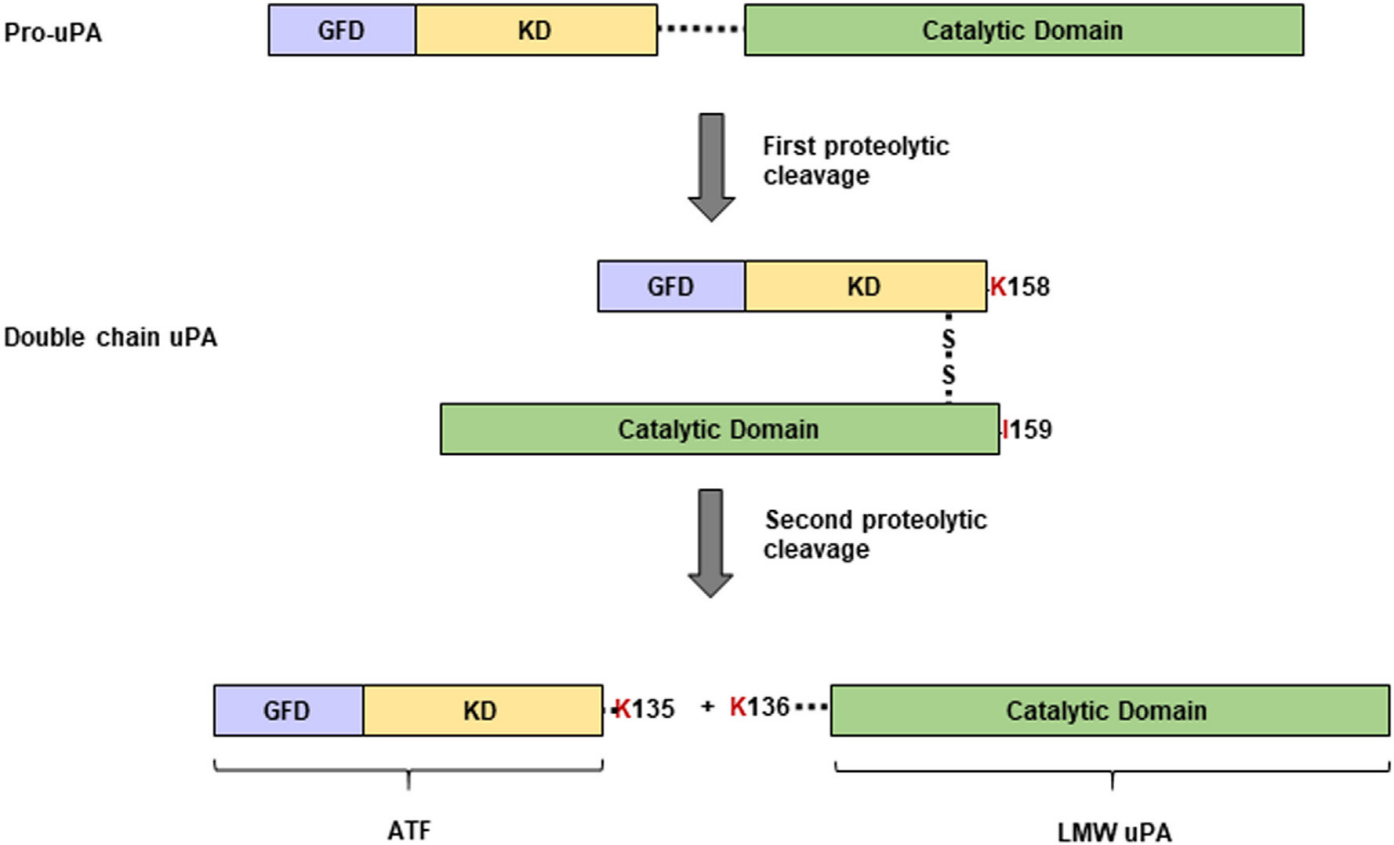
Structure of pro-uPA and uPA. The pro-uPA containing a growth factor domain (GFD), a kringle domain (KD), and a catalytic serine protease domain is secreted as a single-chain precursor that undergoes proteolytic cleavage between the Lys158 and Ile159 peptide bond to generate the two-chain form of uPA. By action of a second proteolytic cleavage, the two-chain form of uPA can be further cut between Lys135 and Lys136 resulting in the formation of an inactive amino-terminal fragment (ATF) as well as a catalytically active low-molecular weight form of uPA (LMW uPA).

uPA is synthesized and released as a single polypeptide chain glycosylated zymogen called pro-uPA (411 amino acids) which consists of three domains: a growth factor domain (GFD) that shares homology with the epidermal growth factor (EGF), a kringle domain (KD), and a serine protease domain (27, 28). The GFD (spanning from 1 to 49 amino acids) and KD (50–131 amino acids) reside at the N-terminus while the catalytic serine protease domain (159–411 amino acids) resides at the “C-terminus.” In between the N-terminal and C-terminal region, there is a linker region (132–158 amino acids) (29). Once the pro-uPA is secreted, it undergoes cleavage of the peptide bond between Lys158 and IIe159 located at the linker region to produce a two-chain form of uPA linked *via* a disulfide bond (30) (Figure 2). Petersen et al. demonstrated that the single-chain pro-uPA is 250-fold less potent to generate active plasmin than the two-chain uPA (31). Different proteases have been reported to function in mediating the cleavage of pro-uPA. Plasmin most effectively converts the pro-uPA into active uPA (30). Among the other proteases that can activate pro-uPA includes cathepsin B and L, nerve growth factor-g, trypsin, kallikrein, thermolysin, and mast cell tryptase (30, 32–34). In addition, proteases such as elastase and thrombin can cause cleavage at different sites of pro-uPA to produce high-molecular weight uPA (30).

Following another round of proteolysis at the peptide bond between Lys135 and Lys136, the two-chain form of uPA can be further cleaved into two parts: (1) a catalytically active low-molecular weight form of uPA having the serine protease domain and (2) an inactive amino-terminal fragment (ATF) that consists of GFD and the KD (28). We have previously shown that the ATF can function as a mitogen (35–38). Due to the presence of GFD, the ATF, the two-chain form of uPA as well as the zymogen pro-uPA can all bind to uPAR at almost similar affinity (28). Binding of uPA to uPAR magnifies its ability to convert plasminogen to plasmin (39). Interestingly, the binding of the catalytically inactive pro-uPA to uPAR can also induce plasmin activation (40). It is speculated that binding to uPAR causes a conformational change in pro-uPA that renders it the ability to activate plasmin even after not being converted into active uPA (40).

uPA has a very high degree of substrate specificity, and plasminogen is its major substrate (41). However, it has been demonstrated that the scatter factor and pro-hepatocyte growth factors and PAI-1 can also be activated by the action of uPA in a manner independent of plasminogen (42, 43). The activity of uPA is regulated by the PAIs and endocytosis (43).

The expression of the gene (*PLAU*) encoding uPA is minimal in normal cells whereas in tumor cells the expression increases several folds. So, what triggers the expression of *uPA* in cancer cells? The answer lies in the fact that the *PLAU* gene expression can be induced by different types of growth factors, hormones, cytokines, as well as by morphological changes of the cells (43). The proximal minimal promoter and the enhancer element located upstream of the transcription start site of the *PLAU* gene are the two regions that regulate its basal and inducible expression, respectively (44). The proximal minimal promoter, associated with the basal expression of the *PLAU* gene, consists of a GC/GA-rich region that resides upstream of the TATA box sequence. This region is recognized by specificity protein 1 (Sp1) and Sp3 transcription factors that aid in the transcription of the basal *PLAU* gene (7). On the other hand, transcription factors Ets1 and Ets2 that binds to the enhancer region located about 2 kb upstream is primarily responsible for the induction of the *PLAU* gene (44). The basal or activated transcription of the *PLAU* gene is regulated by the Jun kinase and mitogen-activated protein kinase signaling pathways (45, 46). Interestingly, the signaling pathways regulating *PLAU* transcription are activated by different types of extracellular stimuli (growth factors, cytokines, etc.) that are frequently elevated during cancer (44). This provides an answer for the elevated expression of the *PLAU* gene seen in cancer. Other *cis-*regulatory sites for nuclear factor (NF)-κB, β-catenin, and T-cell factor (TCF) binding have also been demonstrated to be relevant for *PLAU* gene expression (44).

In addition, DNA methylation-mediated epigenetic mechanisms have been found to be regulating *PLAU* gene expression (47–49) Using surgical biopsy samples from breast cancer patients, our lab has shown that increased DNA hypomethylation at the promoter of the *PLAU* gene correlates with its increased expression in the more aggressive form of the disease (50).

### Urokinase-Type Plasminogen Activator Receptor

**Figure 3:**
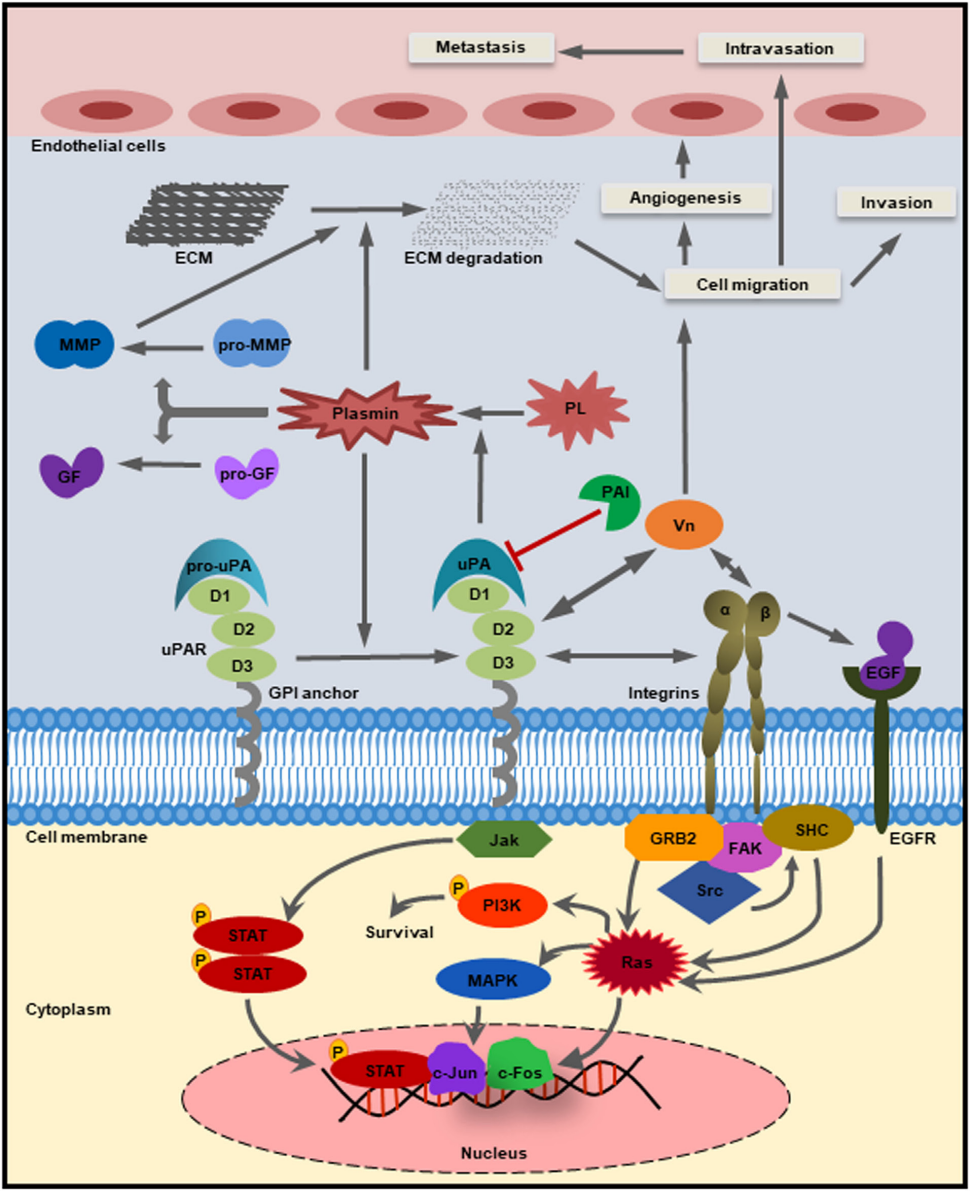
Schematic diagram of the uPA–urokinase-type plasminogen activator receptor (uPAR)-mediated pathways. The glysocylphosphatidylinositol (GPI)-anchored receptor uPAR consisting of three domains (D1, D2, and D3) has the ability to bind the zymogen pro-uPA as well as the active uPA through the growth factor domain. The catalytically active form of uPA then converts inactive plasminogen into plasmin, which in turn can cleave and activate GFs, matrix metalloproteases (MMPs), as well as the extracellular matrix (ECM). The activated MMPs can directly cause the degradation of ECM and thereby release various growth factors. Plasminogen activator inhibitor-1 can inhibit the catalytic activity of both uPA and plasmin. Apart from uPA, uPAR also binds to integrins and other cell surface receptors to activate different intracellular signaling pathways [example: Jak–STAT, PI3K, focal adhesion kinase (FAK), and Rac] and regulates cellular processes such as cell proliferation, survival, migration, invasion, angiogenesis, and metastasis.

The uPAR (CD87) has the ability to localize both pro-uPA and active uPA to the cell surface (39) (Figure 3). It is a single polypeptide chain (313 amino acids) cysteine-rich glycoprotein having three distinct domains (D1, D2, and D3) that are linked *via* two flexible linker sequences (51). The presence of uPAR was first reported in human monocytes and U937 monocytoid cell line (52, 53). This protein is devoid of any transmembrane and intracellular domains, and its C-terminal end is covalently connected to the cell membrane by a glysocylphosphatidylinositol (GPI) anchor (54). Llinas et al. deduced the crystal structure of uPAR and found that the receptor-binding module of uPA binds to the central cavity of uPAR leaving the external receptor surface available for binding with other proteins (55). This implies that the function of uPAR is not only limited to uPA binding and its subsequent activation. Having additional binding site other than uPA helps uPAR to interact with integrins, vitronectin, and different types of transmembrane receptors to facilitate downstream intracellular signaling mediated by well-known effector molecules such as the focal adhesion kinase (FAK), src, and Akt (56) (Figure 3). A number of these effector molecules are widely known to be involved in cancer progression. Different proteases such as matrix metalloproteases (MMPs), plasmin, chymotrypsin, and uPA can cause cleavage of the uPAR protein within the D1/D2 linker region resulting in the formation of a truncated uPAR (19, 54, 57). While all three domains (D1, D2, and D3) of uPAR can bind to uPA, only the D2 and D3 domains can bind to other proteins such as integrins, G protein-coupled receptors (GPCR), and different receptor tyrosine kinases (58–61). In addition, cleavage at the GPI anchor produces a soluble form of uPAR (suPAR) that is present at a very low level in the blood normally (62), but its circulatory level is elevated in cancer (63). The suPAR can activate the G protein-coupled chemotactic receptor FPRL1/LXA4R by functioning as an endogenous chemotactic agonist (64). The uPAR protein can be internalized by the cation-independent, mannose 6-phosphate/insulin-like growth factor II receptor (CIMPR), which facilitates the redistribution of the unoccupied uPAR (65).

The expression of uPAR is elevated in the tumor tissues but not in the surrounding normal tissues, which makes it an attractive therapeutic target (66). More recent evidence indicates that signaling pathways activated by uPAR helps the cancer cells to escape and reduce the cytotoxic effect of anticancer drugs (67). The elevated expression of the gene encoding uPAR (*PLAUR*) in cancer can be regulated by different mechanisms. Transcription factors such as Sp1, NF-κB, TCF, and hypoxia-inducible factor 1α, that are frequently activated by different types of cancer-related signaling pathways, binds to the *cis*-acting elements located upstream of the *PLAUR* gene to trigger its elevated expression in cancer (7). Another important mechanism by which the expression of the *PLAUR* gene gets amplified is through the cooperativity between human epidermal growth factor receptor 2 (*HER2*) and *PLAUR* (68). Using a Real-time quantitative PCR based assay, Pierga et al. first reported on a correlative pattern in the expression of *HER2* and *PLAUR* genes in the disseminated tumor cells from breast cancer patients (69). Later on, Ming et al. showed co-amplification of *HER2* and *PLAUR* when blood and tissue of patients with advanced recurrent breast cancer were analyzed (70). They found that higher the expression of *HER2* in the tumor cells, the higher is the possibility of co-amplification of *PLAUR* in the primary tumors and circulating tumor cells. On the other hand, the *HER2* non-amplified tumors had a significantly lower rate of *PLAUR* amplification. However, the exact mechanism of such co-amplification is yet to be elucidated. Nevertheless, such cooperativity of *HER2* and *PLAUR* suggests the importance of simultaneous targeting of both Her2 and uPAR in breast cancer patients.

### Plasminogen Activator Inhibitors (PAI)

Plasminogen activator inhibitor-1 and PAI-2 are serine protease inhibitors belonging to the serpin family that can cause the neutralization of uPA (71). PAI-1 is more potent than PAI-2 to cause the inhibition of uPA (72). While PAI-1 is predominantly found in the extracellular region, PAI-2 is mainly localized in the cytoplasm for reasons still unknown (73).

Plasminogen activator inhibitor-1, as an inhibitor of PA system, is expected to be associated with anticancer functions by inhibiting the activity of uPA–uPAR complex. Intriguingly, the overexpression of PAI-1 shows a completely opposite effect in cancer patients. Higher expression of PAI-1 promotes tumor growth and as such, it is associated with poor prognosis (73–75). This indicates the fact that PAI-1 has ligands other than the PA system, which takes part in promoting tumor growth (76). In its active conformation, the PAI-1 protein can interact with the integrin binding site on vitronectin and thereby inhibit cellular migration (77). However, PAI-1 interaction binding with uPA (uPA–PAI-1 complex) diminishes its vitronectin binding affinity (77). Both PAI-1 and PAI-2 expressions are altered in cancer. Unlike PAI-1, elevated levels of PAI-2 are associated with the decrease in tumor growth and metastasis (73).

The genes encoding PAI-1 (*SERPINE1*) and PAI-2 (*SERPINB2*) are regulated by different types of growth factors [transforming growth factor beta (TGF-β) and insulin-like growth factor 1], hormones (insulin), and cytokines (tumor necrosis factor α) that typically shows aberrant expression in cancer (7).

## Physiological Role of the uPA–uPAR System

As discussed earlier, the uPA system plays an important role in activating the plasmin from its inactive plasminogen form by proteolytic cleavage. Once activated the plasmin system causes degradation of fibrin, several blood clotting factors, and ECM (78). This, in turn, acts as a homeostatic mechanism in the normal physiologic wound healing. In addition, the components of the uPA–uPAR system are involved in the proteolytic activation of a number of growth factors and cytokines (for example, basic fibroblast growth factor; TGF-β; and interleukin-1 beta) that are involved in myelopoiesis (79, 80).

Degryse et al. demonstrated that the ATF of uPA alone could induce chemotaxis in rat smooth muscle cells through its binding with uPAR (81). Later studies by Mukhina et al. have further streamlined the crucial role of the KD of the ATF in mediating chemotaxis (82). These results suggest that the proteolytic domain of uPA is not required for chemotaxis. Furthermore, antibody-mediated inhibition of uPAR blocked chemotaxis whereas rescue with exogenous uPAR reversed these effects (83). The distribution of uPAR is also different between actively migrating and non-migrating cells. While uPAR is more preferentially distributed on the apical side or the focal contacts on the surface of non-migrating cells (84, 85), however, during migration, it redistributes at the leading edge of the migrating cells (86). In this way, uPAR regulates the concentration and activity of uPA at the required sites on the surface of migrating cells.

It has been shown that the components of the uPA system are expressed in various types of hemopoietic cells (78). More importantly, their levels are altered during the course of infections suggesting the role of the uPA–uPAR system in mediating different types of immune response (87–89). In response to bacterial infection, proinflammatory cytokines such as TNF-α and IL-1β are released, which in turn increases uPA expression and secretion by different types of monocytes, neutrophils, epithelial and endothelial cells (87). Upon its local release, uPA causes the activation of neutrophils, primes them to produce superoxide and potentiates their migration by a pathway that can be either dependent or independent of uPAR (90–93). When uPA is knocked out, it hampered the ability of the mice immune system to recruit neutrophils and macrophages in response to the exposure to *Cryptococcus neoformans* (strain 52D) bacteria, ultimately leading to uncontrolled infection and death of the animals (94). Like uPA, its receptor uPAR also plays an important role in innate immune response through the regulation of cell adhesion, migration in a manner dependent or independent of uPA (88). The uPAR deficient mice also failed to recruit neutrophils and macrophages at the site of bacterial infection (95, 96).

**Figure 4:**
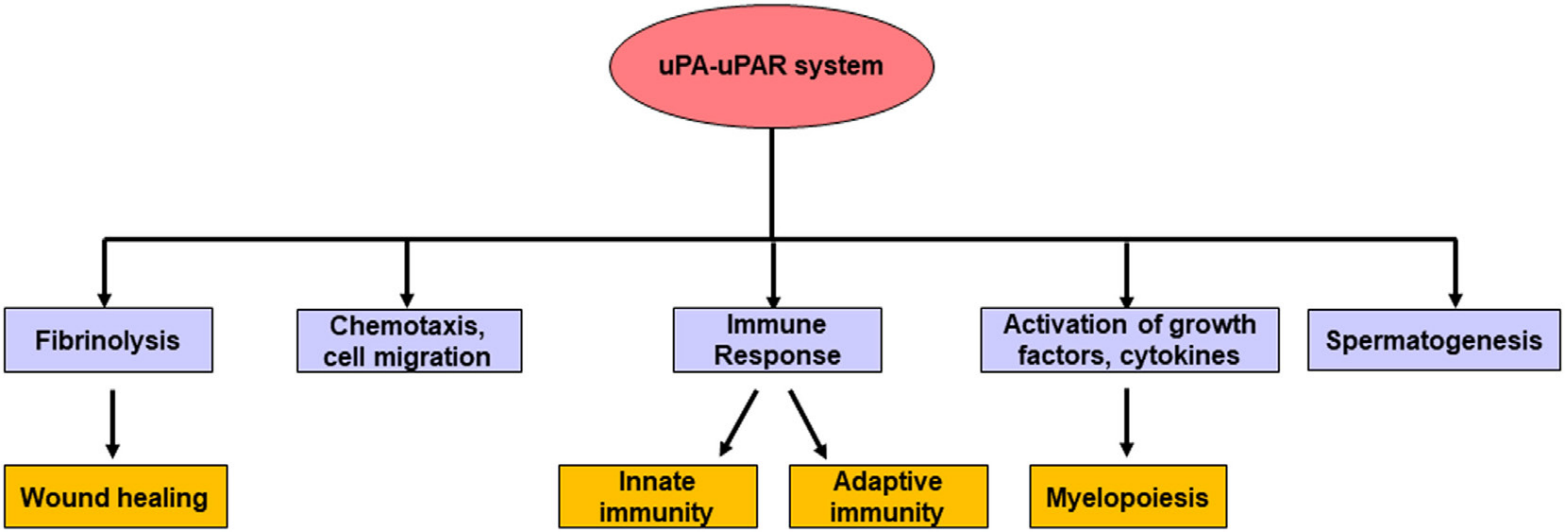
The major physiological roles of the uPA–urokinase-type plasminogen activator receptor (uPAR) system. In normal physiologic conditions, apart from fibrinolysis, the uPA–uPAR system takes part in different biological processes such as the generation of matured myeloid cells, spermatogenesis, chemotaxis, cell migration, wound healing, as well as mediating different types of immune response.

The uPA system has also been implicated in adaptive immunity. The expressions of both uPA and its receptor uPAR are augmented during T-cell activation compared with their levels in resting or naive T cells (97). In uPA-deficient mice, the T helper 1 and 2 (Th1 and Th2) effector lymphocytes lose the ability to respond to pathogen attack (98, 99). On the other hand, blockade of uPAR restricts the migration of leukocytes *in vitro* (100) and also hampers the recruitment of T-cells *in vivo* (101). Collectively, all these studies make it evident that the uPA–uPAR system is at the fringe between fibrinolysis, immune response mechanisms, and pathology (88). In addition, the uPA system is also involved in the male reproductive system where it can enhance sperm mobility, stimulate acrosome reaction, and promote fertilization (102). These district effects of uPA led Qin et al. to demonstrate that targeting uPA may serve as a novel strategy for male contraception (103). These and other physiological functions of the uPA–uPAR system are summarized in Figure 4.

## Function of the uPA–uPAR System in Cancer Progression

The following section summarizes the functional role of the uPA system in different steps of cancer progression.

### Tumorigenesis and Suppression of Apoptosis

The key mechanism that underlies tumorigenesis includes a combination of enhanced cell proliferation and suppression of apoptotic cell death (6). It has been shown that the components of the uPA system can increase cell proliferation through the proteolytic activation of different types of growth factors such as vascular endothelial growth factor (VEGF), EGF, fibroblast growth factor-2 (FGF-2), and TGF-β as well as adhesion molecules such as the α5β1 integrins (6, 104, 105). Several studies have demonstrated the involvement of the uPA–uPAR system during the early stages of tumor formation. For example, the progression of melanoma was impaired in uPA-deficient mice (106). However, inhibition of uPA did not impair the progression of pancreatic cancer in the well-characterized RIP-Tag2 transgenic mice (107), suggesting that the effect of the uPA system in mediating early tumorigenesis is also dependent on cancer types.

Furthermore, the uPA–uPAR system has been implicated in the inhibition of apoptosis (108, 109). Subramanian et al. have shown that RNAi-mediated inhibition of both uPA and uPAR simultaneously triggers apoptosis in breast cancer cells through the upregulation of different caspase proteins (110). Later studies, using targeted antibodies against uPAR (ATN-658) showed increased apoptosis in ovarian cancer cells both *in vitro* and *in vivo* (111). PAI-1, however, has been shown to play a dual role in both augmenting and inhibiting apoptosis. In prostate cancer, PAI-1 expression showed association with increased apoptosis of the endothelial cells of the tumor vasculature (112). In other studies, Chen et al. showed that active PAI-1 causes inhibition of caspase-3 and thereby decreases apoptosis (108). Therefore, PAI-1 is hailed as a “double-edged sword” during apoptosis (113).

### Regulation of the Switch between Dormancy and Tumorigenicity

Cancer dormancy is a stage in cancer progression where the disease remains in an asymptomatic state (114). During tumor dormancy, the cells stop dividing but continue to survive in a quiescent state. Cancer cells may remain at this dormant stage even for decades (114). Once favorable conditions for growth are available, the cells restart proliferation. The switch from a dormant state to a tumorigenic state is one of the underlying causes of disease relapse in clinical settings, which has led to its classification into two broad types, tumor mass dormancy and cellular dormancy. In tumor mass dormancy, the cells continue to divide, but the expansion in tumor size is limited by reduced blood supply or the presence of an active immune system. On the other hand, during cellular dormancy, the tumor growth is halted at the G0/G1 phase of cell cycle. Several mechanisms can regulate the switch from a dormant stage. One such mechanism is mediated by the cross talk between the cancer cells and the surrounding microenvironment through the interaction between uPAR and the integrins (104, 115, 116). In cancer cells, the uPAR–integrin interaction causes the recruitment of FAK and EGFR and thereby induces the mitogenic Raf–MEK–ERK signaling pathway (117, 118). It has been shown that inhibition of uPAR, integrin β1, FAK or EGFR alone, or in combination induces dormancy and thereby results in tumor suppression (115, 118, 119).

### Degradation of the ECM

Extracellular matrix acts as a barrier to confine the tumor cells within its primary site of origin, and proteolytic degradation of the ECM marks a key event during tumor growth, invasion, and metastasis. The proteases that can cause the degradation of the ECM include uPA, plasmin, cathepsins, and different types of MMPs (120). During metastasis, tumor cells cause ECM degradation at various occasions to escape from the primary site of origin to migrate at a distant tissue through the route of the bloodstream. The components of the uPA–uPAR system can breakdown the ECM through the activation of plasmin or the MMPs (6) (Figure 3). Degradation of ECM causes the release of different types of growth factors, which acts as a feedback loop to enhance the expression of different components of the uPA–uPAR system as well as the regulation of various steps of metastasis (7).

### Angiogenesis

Angiogenesis is a biological process by which new blood vessels are developed from the already existing vessels. In tumor microenvironment, the newly formed blood vessels provide oxygen and other nutrients to aid tumor cell growth, invasion, and metastasis (121). The uPA-mediated degradation of the ECM is crucial for the initiation of angiogenesis (7). uPA induces the release of different types of proangiogenic growth factors such as VEGF, FGF-2 that plays a key role in endothelial cell proliferation and invasion (122). The binding of uPAR with vitronectin promotes cell adhesion and migration (123) (Figure 3). uPAR also represses the expression of a key angiogenesis regulator called phosphatase and tensin homolog (*PTEN*) and thereby promotes angiogenesis (124). Loss of function assays using shRNAs against both *uPA* and *uPAR* showed inhibition of angiogenesis signaling by both granulocyte-macrophage colony-stimulating factor (GM-CSF) and VEGF (125, 126). In other studies, inhibition of uPA/uPAR alone or in combination has been shown to repress the expression of Notch-1 (127). This impairs the cross talk of Notch-1 signaling to NF-κB and the PI3K/AKT/mTOR pathways and thereby inhibits invasion and angiogenesis. Whether PAI-1 is proangiogenic or antiangiogenic in cancer is still not clear because of contradictory evidence by different groups (128, 129).

### Cell Adhesion and Migration

Tumor cells need to migrate from the primary site of origin to a distant organ to cause metastasis. This makes cell migration a crucial step during metastasis. Cell migration is dependent on different components of the adhesome, which regulates the attachment and detachment of the cells from the ECM (6). The components of the uPA systems are known to increase cell adhesion and migration during metastatic spread of the tumor cells (130). The role of uPAR has been studied quite extensively in this regard. It is now established that uPAR connects the uPA system to GPCR signaling as well as some other proteins such as cytokeratin 8, α-enolase to regulate cell adhesion, and migration (64, 131, 132). PAI-1 can decrease cell migration by repressing the interaction between vitronectin and αvβ3 integrin (77).

### Cell Invasion and Metastasis

Epithelial–mesenchymal transition (EMT) is a biological process by which polarized epithelial cells are transformed into highly invasive mesenchymal cells with greater migratory capabilities, increased resistance to apoptotic cell deaths as well as enhanced capabilities to produce ECM proteins (133, 134). Elevated uPAR expression during hypoxic conditions activates downstream Akt and Rac1 signaling pathways leading to the promotion of EMT as well as cellular invasion (135). In addition, blocking the expression of uPAR repressed EMT induced by hypoxia. This suggested that uPAR plays a role in EMT. Other studies have shown that antibody-based targeting uPAR also inhibits tumor invasion and metastasis both *in vitro* and *in vivo* (136).

Emerging evidence suggests that tumor cells secrete different types of extracellular vesicles that have pro-metastatic effects (137, 138). Higher levels of uPA and PAI-1 have been detected in the extracellular vesicles of different tumor cells suggesting a possible involvement of the uPA axis in exosome-mediated tumorigenesis and metastasis (139–141). However, detailed studies are warranted in future to verify the phenomena.

## Prognostic Role of the uPA–uPAR System in Cancer

Aberrant expression of the components of the uPA–uPAR system has been detected in a wide variety of cancer (142). This opened up newer avenues to develop therapeutic strategies to attain better clinical outcomes in cancer patients (20, 21). In the following section and Table 1, the role of different components of the uPA–uPAR system in the prognosis of different types of cancer is discussed.

**Table 1:** Selected roles of the uPA–urokinase-type plasminogen activator receptor (uPAR) system in the prognosis of different types of cancers.

Type of cancer	Component	Effect on prognosis	Reference
Breast	uPA and plasminogen activator inhibitor-1 (PAI-1)	Independent prognostic markers for poor relapse-free and overall survival	(145)
	uPAR	Poor prognosis and metastasis during the advanced stages of breast cancer	(69)

Prostate	uPA and uPAR	Increased aggressiveness, postoperative progression, and metastasis	(154)
	PAI-1	Relation to pathological stage, surgical margin status only	(156)
	Soluble form of uPAR (suPAR)	Poor overall survival in patients with prostate cancer	(155)

Ovarian	uPA and PAI-1	Predicts overall survival of advanced staged patients; however, the effect is not consistent between different studies	(161, 162)
	suPAR	Associated with poor survival in preoperative patients; shows stronger prognostic value for assessing the effectiveness of chemotherapy	(157, 160)

Cervical	uPA and PAI-1	Elevated in invasive cervical carcinoma; predicts the risk of lymph node metastasis	(163, 164)
	PAI-2	Elevated in invasive cervical carcinoma	(163)

Endometrial	uPA and suPAR	Elevated in the plasma of endometrial cancer patients	(165, 167)
	uPAR	Correlated with advanced stage endometrial cancer	(166)
	PAI-1	Associated with shorter relapse-free and overall survival	(168)

Soft-tissue sarcoma	uPA	Associated with increased metastasis and recurrence	(169)

Melanoma	uPA and PAI-1	Higher levels in patients is likely to have prognostic significance	(171)

Colorectal	uPA	Prognostic marker for survival and metastasis	(173, 175)
	uPAR, PAI-1, and PAI-2	Higher expression is associated with poor response to therapy	(176)
	suPAR	Higher preoperative level is associated with poor survival	(172)

Lung	uPA, uPAR, PAI-1, and PAI-2	Expression is increased in the tumor tissues of non-small cell lung cancer	(183)
	suPAR	Prognostic marker for lung cancer patients	(181)

Pancreatic	uPA	Increased gene expression is associated with poor survival in pancreatic ductal adenocarcinoma (PDAC)	(185)
	uPAR	Marker to differentiate PDAC and chronic pancreatitis	(186)
	suPAR	Increased urinary level is associated with poor patient outcome in PDAC	(187)
	PAI-2	Gene encoding PAI-2 protein is frequently deleted in PDAC and thereby increase metastasis	(185)

Gastric	uPA	Associated with poor patient outcome.Can be used as a prognostic marker (this is contested by some studies)	(188, 190)
	uPAR	Associated with poor patient outcome	(188)
	PAI-1	Has role as a prognostic marker, which is contested by some studies	(188, 190)

Oral	uPA, uPAR, PAI-1, and PAI-2	Elevated in oral squamous cell carcinoma tumors	(191)
	uPA and PAI-1	Prognostic factor for relapse-free survival	(192)

Esophageal	uPA and PAI-1	uPA/PAI-1 ratio is correlated with invasion	(192)
	uPA	Associated with poor overall survival	(193)
	PAI-2	Protects from local invasion	(193)

Liver	uPA, uPAR, and PAI-1	Higher expression in tumor tissues likely contributes to increased invasion and metastasis	(194)
	uPAR and PAI-1	Correlation with poor prognosis	(194)

Laryngeal	uPA and PAI-2	Independent prognostic factors for survival	(195)

Head and neck	uPA and PAI-1	Expression is increased in the tumors, which is likely to provide prognostic value	(197)
	suPAR	Elevated in the plasma	(198)

Kidney	PAI-1	Influence cause-specific survival	(199)

Bladder	uPA, PAI-1, and PAI-2	Elevated in the tumor samples	(200)

Acute myeloid leukemia	uPAR	Higher expression correlated with aggressiveness of the disease	(201)
	suPAR	Higher level is correlated to chemotherapy resistance	(202)

More than three decades ago, O’Grady et al. demonstrated an elevation of uPA activity in the malignant breast tumors compared with the benign ones (143). Later on, Duffy et al. showed an association between uPA activity in primary breast tumors with tumor size and metastasis (144). Since then a great deal of effort has been made to discover he potential role of uPA and all the components of the uPA system in cancer. It is now evident that the various compartments of the uPA oncogenic pathways are involved in mediating breast cancer progression and metastatic spread. A surprisingly high level of PAI-1 has been implicated in the adverse outcome in breast cancer patients. Using a cohort comprising 2,780 patients with breast cancer, Foekens et al. have demonstrated that the levels of uPA and PAI-1 can be used as independent prognostic markers for poor relapse-free survival as well as of overall survival (145). Both uPA and PAI-1 are categorized as the best available biomarkers after estrogen receptor and HER2 and are among the first to attain level-of-evidence 1 in breast cancer (146, 147). Therefore, the American Society of Clinical Oncology (ASCO) recommended an enzyme-linked immunosorbent assay (ELISA)-based assay to determine uPA/PAI-1 role as a predictive marker for benefit from chemotherapy and as a poor prognostic marker for disease recurrence and survival (148). A follow-up study spanning over 7 years further confirmed the association between invasiveness and increased uPA and PAI-1 levels in patients with breast cancer (149). In recent years, mRNA-based assays such as Oncotype DX (150) and MammaPrint (151) have also shown promising results for predicting outcome in breast cancer. Compared with these assays, the ELISA-based measurement to evaluate uPA and PAI-1 protein is less cumbersome and inexpensive (152). Therefore, it is more practical to use this system for predicting patient outcome in breast cancer. More recently, a prospective randomized multicenter trial called “Chemo-N0” that spanned over a period of 10 years validated the long-term prognostic and predictive impact of uPA and PAI-1 ELISA for the risk assessment as well as treatment decision in patients with node-negative breast cancer (74). Higher level of PAI-2 demonstrated a weak association with favorable outcome in breast cancer (153). Elevated uPAR expression has been linked with poor prognosis and metastasis during the advanced stages of breast cancer (69).

In prostate cancer, high levels of uPA and its receptor uPAR in the plasma correlated with increased aggressiveness, postoperative progression, and metastasis (154). Furthermore, increased suPAR in the circulation showed association with a decrease in overall survival in patients with prostate cancer (155). Using radical prostatectomy specimens from 153 patients, Kumano et al. showed that the expression of uPA and uPAR has a strong correlation with prostate cancer prognosis (156). Furthermore, the expression of PAI-1 expression only showed relation to surgical margin and pathological stage whereas PAI-2 expression did not show any association with prostate cancer prognosis (156).

In ovarian cancer, elevated level of suPAR in the serum from preoperative ovarian cancer patients but not postoperative patients showed association with poor survival (157). The same group has demonstrated a higher level of urinary suPAR in ovarian cancer patients (158). Later studies have found that a portion of the urinary suPAR is present in a cleaved form which is similar to the one in ovarian cancer tissue extracts (159). In another study by Ljuca et al., suPAR, showed a stronger prognostic value for assessing the effectiveness of chemotherapy than carcinoembryonic antigen (CEA) and uPA in FIGO II and III ovarian cancer patients (160). Using an ELISA-based assay from the tumor tissue extracts, Kuhn et al. demonstrated that both uPA and PAI-1 have prognostic significance in predicting overall survival of the patients who have advanced ovarian cancer stage FIGO IIIc (161). However, this observation is not consistent with other studies where uPA and PAI-1 increase did not show any prognostic significance in ovarian cancer patients (162). Further studies are required to demonstrate the exact prognostic role of uPA and PAI-1 in ovarian cancer.

Daneri-Navarro et al. reported an elevated level of uPA, PAI-1 and PAI-2 in the tissue extracts of invasive cervical carcinoma compared with normal tissues (163). Other studies have shown that assessment of uPA and PAI-1 localization in the cervical tissues can predict the risk of lymph node metastasis (164). An increase in the plasma uPA level was demonstrated in patients with cervical as well as endometrial cancer compared with the control group (165). They also reported that the plasma PAI-1 and tPA levels remained unaltered (166). Furthermore, higher levels of plasma suPAR have been identified in endometrial cancer patients than the controls (167). In another study, Memarzadeh et al. found that elevated expression of uPAR in the surgically excised endometrial tissue is positively correlated with advanced stage endometrial cancer (166). Higher levels of PAI-1 protein showed shorter relapse free and overall survival in stage IB and II endometrial cancer patients (168).

Using frozen tumor tissues from 69 patients, Choong et al. detected an association between higher levels of uPA with increased metastasis and recurrence of soft-tissue sarcoma (169). This study indicated a prognostic role of uPA for patients with soft-tissue sarcoma.

In melanoma, a correlation between uPA expression and metastasis was observed in an earlier study using 45 freshly frozen tumors (170). The same study also confirmed the presence of uPAR in one-third of the melanoma tumors (170). Later on, Stabuc et al. reported significantly higher levels of uPA and PAI-1 in melanoma patients compared with normal individuals and further suggested that uPA and PAI-1 might provide prognostic significance in patients with melanoma (171).

In colorectal cancer patients, an elevated preoperative level of suPAR in the plasma demonstrated an association with poor survival (172). In addition, uPA and PAI-1 levels are increased in blood and tissue of the colorectal cancer patients (173, 174). Furthermore, it has been demonstrated that circulating uPA and PAI-1 can serve as better prognostic markers than the commonly used colorectal cancer markers CEA and the gastrointestinal cancer-associated carbohydrate antigen (19–9) (173). Yang et al. suggested that uPA and uPAR can be used as independent prognostic factors for colorectal cancer patient survival, metastasis, as well as therapeutic targets (175). Halamkova et al. showed a statistically significant correlation between the expression of uPAR, PAI-2 protein, and the grade of the colorectal tumor (176). Analysis of the colorectal cancer metastasis in the liver revealed an increase of PAI-1 in the liver metastases compared with primary colorectal carcinomas (177). A number of studies have demonstrated increased levels of uPA, uPAR, and PAI-1 in tissues collected from patients with hepatocellular carcinoma (HCC) as compared with non-cancerous control tissues from the liver (178, 179). In addition, higher expression of these molecules has been linked to HCC metastasis.

Chen et al. demonstrated significantly higher levels of uPA and uPAR in the circulation of lung cancer patients compared with the controls (180). In addition, suPAR has been demonstrated as a prognostic marker for lung cancer patients (181). In small cell lung cancer, the most aggressive form of lung cancer, an association between elevated levels of domain 1 of uPAR (uPAR-DI) in the blood circulation and short overall survival has been demonstrated (182). In non-small cell lung cancer (NSCLC), elevated expression of uPA, uPAR, PAI-1, and PAI-2 proteins were observed in the cancerous tissue compared with the normal/adjacent tissue (183). However, their elevated expression did not show any significant correlation with patient survival.

In pancreatic ductal adenocarcinoma (PDAC), the most common form of pancreatic cancer, the components of the uPA system are frequently altered (184). Recent studies by Harris et al. have demonstrated that the gene encoding PAI-2 (*SERPINB2*) is frequently deleted in PDAC (185). Such deletion may cause an increase in metastasis since PAI-2 is the inhibitor of pro-metastatic uPA. Indeed, when a large cohort of patients with resected PDAC was analyzed, it was found that the expression of the gene encoding uPA (*PLAU*) is significantly increased in these patients. Elevated expression in *PLAU* showed significant association with poorer survival following pancreatectomy. In another study, Chen et al. showed that *PLAUR* can discriminate between PDAC and chronic pancreatitis (CP) with highest accuracy (186). Further studies have demonstrated an elevation of urinary suPAR in PDAC patients compared with the patients with CP (187). Such increase in urinary suPAR in PDAC also correlated with poor patient outcome.

In gastric cancer, uPA and uPAR expression show association with poor patient outcome (188). Both uPA and PAI-1 show prognostic significance in gastric cancer, and their levels are elevated during the advanced stage of the disease (188). Moreover, lower expression of uPA, uPAR, and PAI-1 showed correlation with improved patient survival (189). Using an immunohistochemical method, Luebke et al. could not verify the prognostic significance of uPA and PAI-1 in their study conducted on 105 gastric cancer patients (190). Therefore, further studies are needed to assess the prognostic significance of the uPA system in gastric cancer.

Elevated levels of uPA, uPAR, PAI-1, and PAI-2 are observed in oral squamous cell carcinoma (OSCC) tumors (191). In addition, there is a strong correlation between uPA, uPAR, and PAI-1 expression and aggressiveness of the tumor (191). Hundsdorfer et al. showed that uPA and PAI-1 could be used to assess relapse-free survival in OSCC patients (192).

In esophageal cancer, uPA/PAI-1 ratio shows association with invasive properties of the tumor (192). Shiomi et al. have demonstrated that patients with uPA-positive tumor showed poorer overall survival compared with those with uPA-negative tumor (193). In addition, they have found that less local invasion of esophageal cancer cells in patients with PAI-2 positive fibroblasts suggesting its protective role in tumor invasion.

Zheng et al. showed that the expression of uPA, uPAR, and PAI-1 is significantly higher in HCC cells compared with normal liver tissues and this may contribute increased metastasis (194). Furthermore, uPAR and PAI-1 levels are associated with poor HCC prognosis.

In laryngeal cancer, uPA and PAI-2 have been shown to be strong prognostic factors to determine survival while PAI-1 did not show significant correlation in prognosis (195). It has been shown that the expression of both uPA and uPAR are significantly elevated in laryngeal squamous cell carcinoma compared with the normal peripheral tissues around cancer, which is likely to contribute to increased invasion and metastasis (196).

Strojan et al. found elevated expression of both uPA and PAI-1 in the tumors from the patients with head and neck cancer, which may provide prognostic value to determine invasiveness and metastasis (197). Moreover, significantly elevated levels of suPAR were detected in the plasma of the patients with head and neck cancer, which may provide valuable information for disease prognosis (198).

Ohba et al. reported that PAI-1 has the ability to influence the survival of renal cell carcinoma (RCC) patients (199). Span et al. found that the levels of PAI-1 and PAI-2 but not uPA were significantly elevated in RCC samples compared with normal control tissue (200). On the other hand, the expression of uPA, PAI-1, and PAI-2 were all increased in bladder tumors when compared with normal tissues (200). In acute myeloid leukemia patients, the higher expression of uPAR along with some other morphological characteristics correlated with aggressiveness of the disease (201). Mustjoki et al. found that elevated expression of suPAR showed association with chemotherapy resistance (202). Collectively, these studies have demonstrated a dominant role of the uPA–uPAR in a large number of common cancers, which supports its proposed function as a major player in tumor progression.

## Diagnostic Role of the uPA–uPAR System

**Figure 5:**
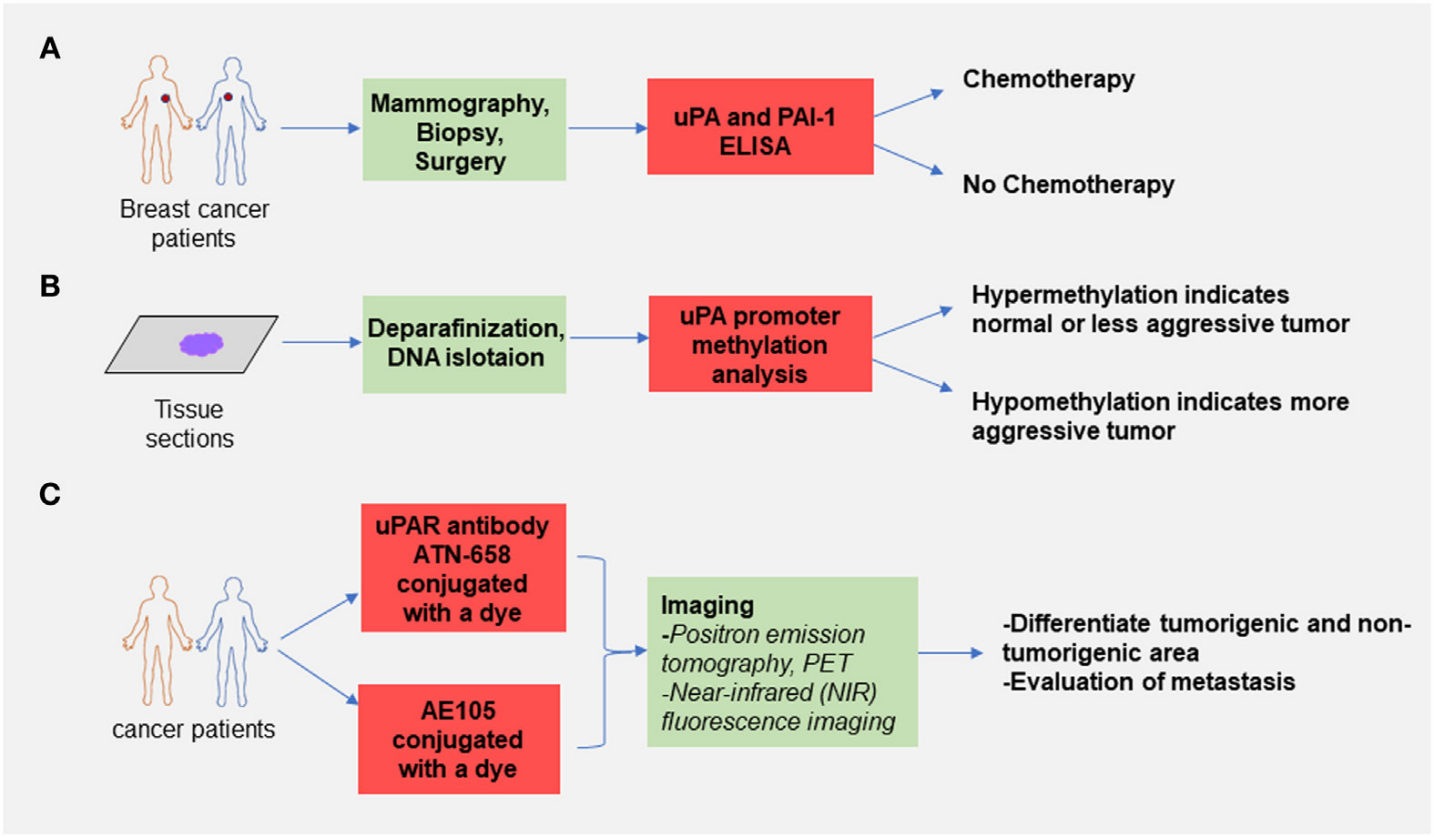
Selected use of the uPA–urokinase-type plasminogen activator receptor (uPAR) system in cancer diagnosis. **(A)** uPA and plasminogen activator inhibitor-1 (PAI-1) enzyme-linked immunosorbent assay can assess the risk of breast cancer recurrence and based on the results obtained from the assay clinicians decide if chemotherapy is needed after surgery. This method is already used by many oncologists. **(B)** Assessment of promoter methylation status of the gene encoding uPA can be used to predict the aggressiveness of the primary tumor. It has been shown that the CpG sites on the uPA promoter are hypomethylated in high-grade tumors compared with the control and less aggressive tumors. This method can be used to determine the invasiveness of cancer. **(C)** Conjugation of a labeled dye with antibodies (ATN-658 for uPAR) or peptide targeting uPA–uPAR interaction (AE105) can distinguish between the tumorigenic and non-tumorigenic area, and this method showed great diagnostic potential in several known cancers.

Since the uPA–uPAR system plays a crucial role in the progression of cancer, a plenty of efforts have been made to develop diagnostic modalities targeting this axis (203–205). Figure 5 contains a brief summary of some of the promising use of the uPA–uPAR system in cancer diagnosis.

### uPA and PAI As Diagnostic Biomarker

Using ELISA, Jänicke et al. were the first to determine the amount of uPA protein in breast cancer tissue (206). They found that the uPA antigen is elevated in the primary breast tissues, which correlated with poor breast cancer prognosis of the patients. A similar association was also reported for PAI-1 by the same group in 1991 (207). Later on, the ASCO recommended uPA and PAI-1 biomarker testing for breast cancer risk assessment, and also to decide on the appropriate adjuvant chemotherapies to be given to the patients (148).

Enzyme-linked immunosorbent assay-based methods are still considered as the clinically relevant system for assessment of uPA and PAI-1 in the context of breast cancer outcomes. As such commercial ELISA kits for uPA/PAI-1 have been developed. FEMTELLE^®^ is one such kit that is used to assess the risk of recurrence in primary breast cancer and helps to predict if the chemotherapy is beneficial after surgery (208). However, the main problem with all the ELISA-based assays is the need for fresh or fresh-frozen tissue samples (209). Hence, the possibilities of using some other methods including histological analysis that do not require fresh or fresh-frozen tissue samples have also been investigated.

Several groups have assessed uPA and PAI-1 mRNA expression to diagnose cancer (210, 211). The main advantage of using mRNA is that it can be extracted from the formalin-fixed tissues unlike the ELISA-based assays. However, there are discrepancies between studies on the effect of uPA and PAI-1 mRNA expression in cancer diagnosis (210–212). More research is needed to come up with a concrete conclusion.

Another interesting avenue that employs the assessment of uPA to diagnose cancer is through the analysis of alterations of the methylation status of the promoter DNA. The major advantage of this method compared with the other techniques is the fact that DNA is more stable and can be isolated from the formalin-fixed, paraffin-embedded samples (209). Aberrant DNA methylation is seen in almost all cancer. Both hypermethylation-mediated inactivation of tumor suppressor genes and hypomethylation-mediated activation of pro-metastatic genes are key characteristics of the cancer cells, which makes DNA methylation as a suitable diagnostic and therapeutic target (213). More than a decade ago, our lab demonstrated that there is an inverse correlation between uPA expression and promoter methylation for different grades of breast cancer (50). We demonstrated that the percentage of CpG methylation at the *uPA* promoter is decreased with the progression of breast cancer (50). As such uPA promoter methylation status can be used as an early detection marker. Gao et al. have demonstrated a similar inverse correlation between PAI-1 promoter methylation and gene expression in cancer (214).

### uPAR As an Imaging Agent in Malignancy

An important avenue where much attention has been deployed is the development of non-invasive imaging/diagnostic agents targeting the uPA system. In particular, imaging strategies targeting uPAR has been used in different types of cancer (204, 205). Using rodent models of breast and prostate cancer, we have shown that uPAR is a viable imaging target for cancer diagnosis (215). Briefly, an ^125^I-labeled anti-rat uPAR antibody was injected into animals bearing prostate and breast cancer and the uptake of radiolabel in primary tumors as well as some of the common sites of metastasis including the liver, lung, spleen, and lymph nodes was determined. On the other hand, control animals with no tumors or the tumor-bearing animals injected with ^125^I-labeled control IgG antibody showed a very minimum radioactivity levels suggesting that uPAR as a diagnostic/imaging target for cancer progression and metastasis. Since then a plethora of studies have been done to validate and improve the diagnostic potential of uPAR in different types of cancer. Among these, well-known examples are peptide antagonist targeting uPA–uPAR interaction (AE105) and monoclonal antibodies against uPAR (ATN-658). Both these molecules have been used for multimodal imaging for cancer diagnosis. The main idea behind the multimodal imaging is to conjugate a single target to either a radionuclide or a near-infrared (NIR) fluorescent dye that can distinguish the tumor tissue from the surrounding non-tumorigenic area before [positron emission tomography (PET) or single-photon emission computed tomography] or during (NIR fluorescence imaging) surgery (216).

AE105 is a smart peptide composed of nine amino acids and is used for intraoperative guidance at the time of surgery as well for the evaluation of metastasis (204, 217). On the other hand, different classes of antibodies such as ATN-658 (targeting uPAR), ATN-291 (targeting uPA) for cancer imaging has been reported (205, 218). The ATN-658 is a humanized antibody that is currently being used by us and others.

While both AE105 and ATN-658 have antitumor activity, however, they exhibit different imaging timeframes. Since it is a small molecule of 1 kD, the imaging timeframes of AE105 are usually within several hours due to short half-life (216, 217). On the other hand, ATN-658 has much longer half-life in the serum, which enables the imaging timeframes to go up to days (64, 216). AE105 cannot target uPAR when it is already attached to uPA and therefore the imaging intensity of the tumors targeted with AE105 peptide-based agents may not always provide information on the actual levels of uPAR. On the other hand, ATN-658 binding to uPAR is independent of the uPA–uPAR interaction, which helps to provide more accurate diagnostic information. Recently, a phase 1 clinical trial using AE105 has been completed in patients with breast, prostate, and bladder cancers (217). In these studies, AE105 was conjugated to the organic macrocyclic chelator 1,4,7,10-tetraazacyclododecane-1,4,7,10-tetraacetic acid (also called DOTA) and then labeled with ^64^Cu for PET imaging and found that administration of the agent is safe in cancer patients with a favorable biodistribution and stability. The clinical trial for ATN-658 imaging is expected to start soon by us and others. Taken together, these studies have shown great promise for the use of uPAR as a diagnostic/imaging target in malignancy.

## Therapeutic Targeting of the uPA–uPAR System

Many studies spanning more than two decades have made attempts to target the uPA system to block cancer (219, 220). Some of the most notable ones are listed below.

### Inhibition of Proteolytic Activity of uPA

The earliest attempts to block uPA activation focused on the development of agents to inhibit its catalytic activity. Ossowski et al. used an antibody-based approach to block the enzymatic activity of uPA that was able to block local invasion but failed to inhibit distant metastasis *in vivo* (221). Later studies using small molecule inhibitors proved to be more effective in achieving the inhibition of the enzymatic activity of uPA. By modifying the chemical structure of amiloride, Towle et al. were able to develop a novel class of inhibitors of uPA known as the 4-substituted benzo(b)thiophene-2-carboxamidines (222). Two compounds belonging to this family known as B-428 and B-623 were able to inhibit uPA activity with median inhibition concentration (IC_50_) values of 0.32 and 0.07 µM and inhibitory constant (*K*_i_) values of 0.53 and 0.16 mM (222). We have previously shown that use of a small molecule inhibitor called B-428 can reduce prostate cancer growth and metastasis *in vivo* (223). Further work in our lab has shown that B-428 has an additive effect in repressing breast cancer growth and metastasis when used in combination with tamoxifen (224). Other inhibitors of uPA activity have also been used and some of them have shown very promising results in the clinical trials. For example, MESUPRON^®^ (also known as WX-671) and its pro-drug WX-UK1 have shown promising results in the clinical trials for the treatment of different types of solid tumors (225–227).

### Inhibitors of the uPA–uPAR Interaction

More than 20 years ago, Crowley et al. showed that competitive removal of uPA from its receptor by the action of a catalytically inactive analog (Ser 356 → Ala) inhibits prostate cancer metastasis *in vivo* (228). Since then many inhibitors have been developed to cause an interruption in the uPA–uPAR binding (229–231).

Another approach to inhibit the uPA–uPAR interaction functions by blocking the linker peptide of uPA. It has been shown that phosphorylation at Ser138 residue of uPA, which is located in the linker region, causes inhibition of its uPAR-dependent myelomonocytic adherence and motility (232). Our group was the first to demonstrate that an 8-mer (acetyl-KPSSPPEE-amino) non-competitive capped peptide antagonist of the uPA–uPAR interaction called Å6 can inhibit tumor growth and metastasis *in vivo* (22). In addition, we showed that Å6 enhances the anticancer effects of hormone therapy when used in combination with Tamoxifen in a rat syngeneic model of breast cancer (233). Boyd et al. showed that Å6 treatment prolonged survival of mice bearing prostate cancer cells and also reduced lymph node metastases *in vivo* (234). In a xenograft model of glioblastoma, the combination of Å6 with standard mode of therapy, i.e., cisplatin showed enhanced antitumor and antiangiogenesis effect than either cisplatin or Å6 alone (235). Piotrowicz et al. have showed that Å6 can also inhibit B16-F10 melanoma cell migration and metastasis *in vivo* (236). Taken together, the results from these studies accelerated the clinical evaluation of using Å6 for different types of cancer. Phase 1 and 2 clinical trials using Å6 have shown a promising efficacy as well as safety profile (237). However, it has shown a modest anticancer effect for the treatment of gynecologic cancers in Phase 2 clinical trials (238).

### Targeting uPAR in Malignancy

In this age of targeted therapies and precision medicine, the use of antibodies directed against a specific metastatic or oncoprotein (for example, HER2 in breast cancer) has shown a great promise. We and others have also used a number of antibodies targeting uPAR to block its interaction with uPA or the integrins (41, 136, 215, 220). More than a decade ago, our group showed that a polyclonal antibody raised against rat uPAR can cause inhibition of breast cancer growth and metastasis *in vivo* (215). Later on, by using a monoclonal antibody (ATN-658) raised against human uPAR protein, our group showed the ability of ATN-658 to block prostate cancer cell proliferation, invasion, and metastasis (136). The main advantage of using ATN-658 is that it can bind to uPA-occupied uPAR unlike many other predecessor antibodies that targeted uPAR (41). Moreover, it has shown anticancer activity in a wide range of cancers (111, 136, 239). Taken together, the preclinical results showed great promise for the use of ATN-658 in clinical trials for further evaluation of its safety and efficacy in a human population.

### Transcriptional Repression of the Components of the uPA System

Various techniques aiming to repress the expression of the uPA system have been evaluated that includes the use of antisense oligonucleotides or RNA interference (RNAi), ribozymes, etc. Antisense oligonucleotides targeted against uPAR have shown to cause a reduction in cell proliferation, invasion, and metastasis in different types of cancer (240–242). RNAi-mediated inhibition of both *uPA* and *uPAR* in human glioma cells increased apoptosis as well repressed the PI3K/AKT pathway (243). A specific ribozyme targeting *uPAR* mRNA has been used to disrupt uPAR translation in human osteosarcoma cells *in vitro* (244).

We have shown a DNA hypermethylation-mediated inhibition of *uPA* in different types of cancers both *in vitro* and *in vivo* (245, 246). Pakneshan et al. have shown that the *uPA* promoter is hypomethylated in patients with aggressive breast cancer (50). By using the universal methyl group donor *S*-adenosylmethionine (SAM), we could reverse the hypomethylated state of *uPA* and thereby cause a decrease in the expression of *uPA* (245). SAM treatment decreases tumor cell proliferation, invasion, and metastasis (247). It has also been shown that SAM causes transcriptional repression of a number of pro-metastatic genes apart from *uPA*. This is interesting because SAM is an approved dietary supplement that can function as a chemopreventive agent, and SAM-mediated repression of *uPA* would help the patients to take fewer amounts of the agents targeted against the uPA system and thus reduce the probability of any potential side effects. Apart from SAM, different types of growth factors, hormones, and cytokines have been shown to repress *uPA* and *uPAR* expression (248, 249).

### Targeting PAI-1 Using RNA Aptamers and Peptide Inhibitors

RNA aptamers are oligonucleotides that are capable of folding into complex structures and then bind to various macromolecules with high affinity and selectivity (250). Two RNA aptamers WT-15 and SM-20 have been shown to disrupt the PAI-1 and vitronectin interaction without causing any effect on the ability of PAI-1 to inhibit uPA (251). It has been shown that the vitronectin and PAI-1 interaction promotes metastasis by causing the detachment of tumor cells from the ECM (252). Therefore, by repressing this interaction, the RNA aptamers WT-15 and SM-20 show an antimetastatic effect in cancer. Another way to inhibit PAI-1 is using peptides such as paionin-4-D1D2 that can stimulate PAI-1 conversion into its latent form (253).

### Use of Toxins and Nanobins Combine with uPA Agents

Conjugation of cytotoxic drugs with agents directed against various components of the uPA system has been evaluated as a therapeutic strategy. For example, conjugation of DOTA and 213-Bi with the GFD of uPA has shown to cause a cytotoxic effect on ovarian cancer cells expressing uPAR (254). Several groups have targeted the ATF region of uPA by conjugating with a toxin. For example, conjugation of the ATF with the catalytic component of the diphtheria toxin (DTAT) reduced glioblastoma tumor growth (255, 256). Huang et al. demonstrated the cytotoxic effect of DTAT in a mouse model of human metastatic NSCLC to the brain (257). They have shown that treatment with DTAT significantly prolonged survival in the treated animals compared with the controls. In another study, a truncated form of *Pseudomonas* exotoxin (PE) conjugated with ATF showed cytotoxic effects in cell lines from different types of cancer (258).

In the recent years, some researchers have tried to combine the agents targeting the components of uPA system with the nanobin technology. Nanobins are liposomal nanoparticle drug encapsulation and formulation system by which more precise targeting of the tumor tissue can be done (259). The major advantages of the nanobins include increased circulation half-life, lesser off-target effects, and more specific delivery of the therapeutic payloads into the tumor cells (260). Nanobins can be easily conjugated with a target-specific antibody to potentiate a highly specific antibody-directed cellular cytotoxicity (260, 261). The uPA antibody ATN-291 has been conjugated with nanobins, which enhanced its internalization by the uPA or uPAR expressing tumor cells compared with the cells that do not express uPA or uPAR (58, 260). In addition, nanoparticles conjugated with ATF to target uPAR have been demonstrated by several groups (58). For example, Yang et al. conjugated iron oxide nanoparticles to ATF (ATF-IO) for delivery to breast cancer cells expressing uPAR and further suggested that ATF-IO nanoparticles can be potentially used as molecularly targeted, dual-modality imaging agents (262). In another study, Abdalla et al. have conjugated IO and noscapine to ATF and demonstrated that these conjugates have cytotoxic effects on prostate cancer cells (263). In a recent study, Carriero et al. used a Retro-Inverso (RI) approach to block the interaction between uPAR and G protein-coupled formyl peptide receptors and observed a significant reduction in tumor growth and decreased the number of circulating tumor cells and pulmonary metastases in immunocompromised mice injected with sarcoma cells (264). Further studies are warranted in this attractive area of uPAR-targeted therapeutic agents in different types of cancer.

## Conclusion and Future Perspectives

Research over the past 30 years has demonstrated that the uPA–uPAR axis has a pleiotropic effect in different stages of cancer. Abnormalities in the levels of the components of the uPA–uPAR are frequently observed in a number of malignancies. Further mechanistic studies identified it as an oncogenic pathway for tumor growth proliferation and metastatic spread. Therefore, it has become a target for cancer prognosis, diagnosis, and therapeutic interventions. The preclinical data by us and others have demonstrated that targeting this axis shows association with tumor suppression and reduction of metastasis. In the next few years, the therapeutic and diagnostic potential of the uPA–uPAR system will continue to be evaluated for different types of cancer in clinical settings in combination with chemotherapeutic agents, targeted therapies and novels agents that alter tumor cell growth, signaling, and immune mechanism. Based on the strong preclinical data, these studies are expected to provide better diagnostic and therapeutic options for the patients to improve cancer-associated morbidity and mortality.

## Author Contributions

All authors listed have made a substantial intellectual contribution and approved it for publication.

## Conflict of Interest Statement

The authors declare that the research was conducted in the absence of any commercial or financial relationships that could be construed as a potential conflict of interest.
